# 3-Amidinophenylalanine-derived matriptase inhibitors can modulate hepcidin production in vitro

**DOI:** 10.1007/s00210-019-01743-x

**Published:** 2019-10-28

**Authors:** Erzsébet Pászti-Gere, Gergely Szombath, Michael Gütschow, Torsten Steinmetzer, András Székács

**Affiliations:** 1 Department of Pharmacology and Toxicology, University of Veterinary Medicine, Budapest, Hungary; 2 Pharmaceutical Institute, University of Bonn, Bonn, Germany; 3 Institute of Pharmaceutical Chemistry, Philipps-University Marburg, Marburg, Germany; 4 Agro-Environmental Research Institute, National Agricultural Research and Innovation Centre, Budapest, Hungary

**Keywords:** Matriptase inhibitors, Hepatocytes, Hepcidin, Hydrogel matrix, Extracellular ROS

## Abstract

Matriptase-2 (MT-2) is a type II transmembrane serine protease and predominantly attached to the surface of hepatocytes. MT-2 decreases the production of hepcidin, a key regulator of iron homeostasis. In this study, the effects of four 3-amidinophenylalanine-derived combined matriptase-1/matriptase-2 (MT-1/2) inhibitors (MI-432, MI-441, MI-460, and MI-461) on hepcidin production were investigated in hepatocyte mono- and hepatocyte-Kupffer cell co-cultures. In MI-461-treated cell cultures, the extracellular hydrogen peroxide contents and the interleukin-6 and -8 (IL-6 and IL-8) levels were determined and compared to controls. Hepcidin overproduction was observed in hepatocytes upon treatment with MI-432, MI-441 and MI-461 at 50 μM. In contrast, extracellular hydrogen peroxide levels were not elevated significantly after matriptase inhibition with MI-461. Furthermore, MI-461 did not induce increases in IL-6 and IL-8 levels in these hepatic models. A model of the binding mode of inhibitor MI-461 in complex with MT-2 revealed numerous polar contacts contributing to the nanomolar potency of this compound. Based on the in vitro data on hepcidin regulation, treatment with MI-461 might be valuable in pathological states of iron metabolism without causing excessive oxidative stress.

## Introduction

Hepcidin is mainly produced in the liver and acts as a key regulator of iron homeostasis. It prevents excessive iron from entering the blood circulation via inhibiting the absorption of nutritional iron from enterocytes and suppressing the release of iron. When the blood iron concentration is high, hepcidin production in the liver becomes elevated and contrarily, in the case of the iron shortage, the hormone secretion decreases significantly leading to an enhanced iron transport (Ganz and Nemeth 2012).

Thus, hepcidin prevents iron overload (Knutson et al. 2005) which could lead to the formation of reactive oxygen species (ROS) or to severe damages to subcellular components such as electron transport chain of respiration or mitochondrial DNA content. Furthermore, the rapid iron-lowering ability of hepcidin seems to be a part of the conserved evolutionary responses against pathogenic infections, which restricts the access of bacteria to iron sources essential for their further survival (Ganz and Nemeth 2012; Gao et al. 2009). Hepcidin synthesis in the liver is upregulated by increasing levels of plasma iron supported by the fact that iron-transferrin saturation can stimulate transcription of the hepcidin encoding hepcidin antimicrobial peptide (HAMP) gene.

In the case of anemia of inflammation or in the case of prolonged conditions, anemia of chronic disease, macrophages do not release appropriate amount of iron into the bloodstream due to the antiinfectious function of hepcidin causing manifested systemic iron shortage (Ganz and Nemeth 2012). Several studies were performed to establish a relationship between hepcidin secretion and pathogen-provoked inflammatory responses. Administration of lipopolysaccharide (LPS) could lead to elevation in hepcidin-encoding mRNAs in hepatocytes and in mice (Pigeon et al. 2001). Similar results were detected in mice exposed to *Candida albicans* and in human hepatoma cell lines infected with influenza A virus (Armitage et al. 2012). IL-6 seemed to be a key mediator among pro-inflammatory cytokines in the initiation for hepcidin overproduction in LPS-triggered inflammation, as application of IL-6 neutralizing antibodies could significantly decrease the synthesis of hepcidin mRNA. In accordance, IL-6 applied in infusions could enhance hepcidin amounts detected in human urine within few hours (Nemeth et al. 2004).

Hepcidin level is reduced in hemolytic anemia and anemias with ineffective erythropoesis. Hypoxia can trigger the erythropoiesis via erythropoietin (EPO) synthesis in kidney and liver resulting in significant suppression of hepcidin; however, the main factor in hepcidin regulation is the iron load of serum transferrin, and not the effect of EPO (Piperno et al. 2011). Hepatic oxidative stress as a consequence of excessive alcohol consumption or viral infections can inhibit hepcidin production and lead to iron overload which can further deteriorate the already existing pathophysiological conditions (Fujita et al. 2007; Harrison-Findik 2007).

Type II transmembrane serine proteases (TTSPs) modulate proteolytic processes on cell surfaces. The TTSPs can be divided into four subfamilies, the matriptase, the hepsin/TMPRSS, the corin, and the HAT/DESC family. All of these proteases possess a cytoplasmic N-terminal segment, followed by a transmembrane domain, a middle multidomain-like stem region of variable lengths, and a C-terminal trypsin-like serine protease domain (Bugge et al. 2009). Matriptase-2 (MT-2 or TMPRSS6) similarly to matriptase-1 (MT-1) is bound to cell membrane, and they are both capable of degrading extracellular matrix (ECM) proteins. MT-2 is mainly found in human adult and embryonic liver.

Moreover, an extrahepatic occurrence of MT-2 encoding mRNAs was detected in kidney and in uterine of mice (Hooper et al. 2003). MT-2 takes part in the suppression of hepcidin production via cleavage and decomposition of hemojuvelin (HJV). In the liver, bone morphogenetic protein (BMP) pathway acts as one of the key regulators for hepcidin transcription thus for the iron metabolism. These findings were supported by the fact that hepcidin responses to the acute and to the chronic iron loading were impaired upon loss of these molecules (Babitt et al. 2006; Huang et al. 2005; Corradini et al. 2011). It was also proven that the catalytic domain together with the stem region of MT-2 is necessary for the effective inhibition of hepcidin expression. TMPRSS6^−/−^ mice have a dysfunctional MT-2 due to a deficiency in the catalytic ectodomain, and it was observed that iron deprivation itself did not induce elevated iron absorption by enterocytes in mice with mutant MT-2 (Du et al. 2008). TMPRSS6^−/−^ mice with MT-2 deficiency suffered from microcyter hypochrom anemia accompanied by alopecia, hair follicular dystrophy, and hyperkeratosis. This condition could be improved with parenteral iron-dextran treatment suggesting the potential impact of MT-2 on iron homeostasis (Folgueras et al. 2008). Accordingly, inhibition of MT-2 has been proposed as a new therapeutic opportunity to treat iron overload diseases (Sisay et al. 2010; Stirnberg and Gütschow 2013; Maurer et al. 2012).

In human patients and mice with iron-refractory iron deficiency anemia (IRIDA), a genetic impairment of MT-2 was observed to cause microcyter, hypochrom anemia with low transferrin saturation (Finberg et al. 2008). Based on these findings, it can be suggested that damage in the catalytic domain of MT-2 can affect adaptation ability when iron sources in the body are lowered both in humans and in mice, and excessive hepcidin synthesis can facilitate sequestration of iron into macrophages and decreased iron absorption.

**Fig. 1:**
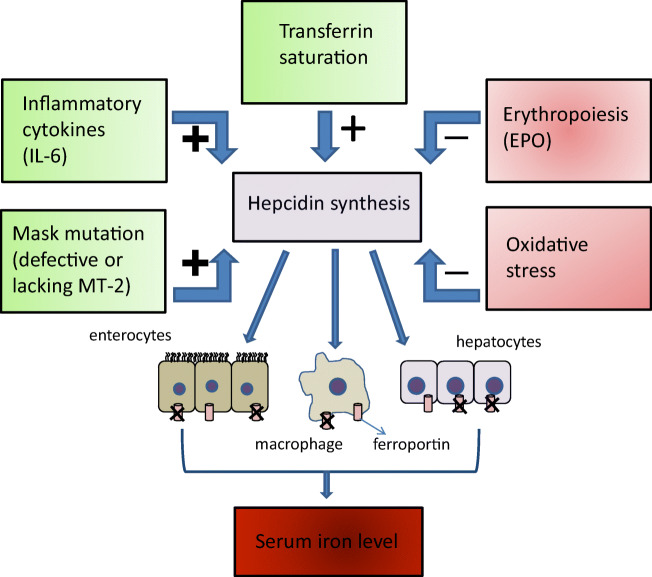
The iron level in the blood is dependent on hepcidin production maintained by positive regulators, like transferrin saturation, interleukin-6 (IL-6), and defective matriptase-2 (MT-2) (green boxes) and negative regulators such as erythropoietin (EPO), and oxidative stress (light red boxes). The iron homeostatic processes involve interplay between macrophages, hepatocytes, and intestinal epithelial cells. Stimulatory effects in hepcidin synthesis are indicated with + and the inhibitory factors of the production is labelled with − sign. Increased hepcidin levels result in reduced serum iron levels due to the inhibition of ferroportin, and vica versa

The positive and the negative regulators of the hepcidin production and its effects on iron homeostasis are summarized in Fig. 1. The extracellular trypsin-like serine protease domain at the C-terminus of MT-1 and MT-2 can be inhibited by sulfonylated 3-amidinophenylalanine-derivatives, by various cyclic peptidic inhibtors (Quimbar et al. 2013), short linear peptides with a C-terminal arginylketone moiety (Colombo et al. 2012) or dipeptide derivatives containing C-terminal decarboxylated arginine mimetics (Sisay et al. 2010), or other zwitterionic amino acid derivatives (Pászti-Gere et al. 2015) such as glyphosate, which is likely to disrupt transferrin-based iron uptake (Samsel and Seneff 2016). Moreover, surfactant polyethoxylated tallow amines were also found to inhibit matriptases possibly through membrane disruption (Pászti-Gere et al. 2015). An inhibition of MT-1 and MT-2 is also achieved by sulfonylated 3-amidinophenylalanine derivatives (Hammami et al. 2012). The most potent analogues of this inhibitor-type contain an N-terminal dichloro- or dimethoxy-biphenyl-3-sulphonyl group and inhibit MT-1 in the low one-digit nanomolar range, whereas the best inhibition constants for MT-2 are close to 100 nM (Hammami et al. 2012; Pomothy et al. 2016). However, the effects of these combined MT-1/2 inhibitors on the compensation for iron homeostatic imbalances caused by dysregulated hepcidin production have not been elucidated yet.

A reliable mono- or co-culture model in an environment which ensures appropriate cell adhesion and maintains cell-cell contacts is needed to investigate the influence of drug candidates in vitro and to predict their potential hepatic effects in vivo. The assembly of hepatocytes into 3D structures contributes to the maintenance of hepatocyte function to greater extent over conventional monolayer cell systems. Hydrogel is one of the support materials for the currently available 3D environments offering an excellent basis for the development of the desired 3D cell structure (Bachmann et al. 2015). Recently, hydrogels have been widely used for pharmacological investigations, toxicity, or controlled drug release studies (Huh et al. 2011; Haycock 2010; Lee et al. 2008; Almany and Seliktar 2005; Pongrácz and Bartis 2011; Bhattacharya et al. 2012; Khademhosseini and Langer 2007).

The aim of our study was to screen the pharmacological effects of 3-amidinophenylalanine-derived protease inhibitors on iron homeostasis via hepcidin modulation in an in vitro hepatic model such as hepatocyte mono- and hepatocyte-Kupffer co-cultures. After treatment with compound MI-461, a profiling of its IL-6 and IL-8-regulating and oxidative stress-inducing properties was performed to elucidate and to predict the safety of this inhibitor. Special emphasis was laid on evaluation and comparison of the data obtained from 2D and hydrogel-assisted hepatic cell mono- and co-cultures exposed to MT-1/2 inhibition. Moreover, based on a previously described homology model of MT-2 (Sisay et al. 2010; Maurer et al. 2012), a reasonable binding mode of inhibitor MI-461 within its active site was predicted.

## Materials and methods

### Isolation and cultivation of hepatocytes and Kupffer cells

Hepatocytes and hepatocyte-Kupffer cell co-cultures were freshly prepared from 15 kg weighing male pigs of the Hungarian Large White breed (obtained from Dunahyb Ltd, Fadd, Hungary) (Mátis et al. 2016). Hepatocytes and Kupffer cells were isolated by multi-step perfusion and collagenase-mediated enzymatic digestion of the caudate lobe of the liver. All animal experiments were carried out in accordance with the ethical guidelines for the care and use of laboratory animals. The study was authorized by the Animal Welfare Committee of the University of Veterinary Medicine in Budapest, Hungary. Hepatocytes were sedimented and purified by a revised low-speed centrifugation (50×*g*, 75 s), and Kupffer cells were obtained from the supernatants by Percoll gradient centrifugation (500×*g*, 20 min). As non-parenchymal cells can attach faster, Kupffer cells were seeded first on previously collagen-coated (Type I, 10 μg/cm^2^) 12-well Costar TC6 cell culture dishes (Corning International, Corning, NY, USA) and on 96-well microplates, the latter used for MTS assays on cell viability. Hepatocytes in mono-cultures (Hep) or hepatocytes and Kupffer cells (HepK) were seeded in cell ratio 6:1 (hepatocyte to Kupffer cell). The prepared mono- or co-cultures were incubated under humid atmosphere at 37 °C using 10% CO_2_, and culture medium was exchanged 6 h after seeding. Culturing of cells was performed in Williams’ Medium E, supplemented with penicillin–streptomycin (1%), glutamine (2 mM), FBS (5%), and insulin (20 IU/ml). Fetal bovine serum (FBS) was applied during the first 24 h of culturing. Confluent mono- and co-cultures suitable for the further examinations were obtained after 24-h incubation. Cell lysate protein content was spectrophotometrically determined with Bio-Rad Bradford protein assay (Bio-Rad Laboratories, Inc., Hercules, CA, USA) at 595 nm with bovine serum albumin as a standard.

### Hydrogel-based cell culturing

Lyophilized HydroMatrix (Sigma-Aldrich, Saint Luis, Montana, USA) was used at 0.25% concentration for hydrogel preparation. Hepatocytes and Kupffer cells were added to the hydrogel-assisted matrices at the physiological pH. The culturing of Hep and HepK cells was carried out in Williams’ E medium on hydrogel-covered 6-well polyester membrane inserts (Costar Transwell, surface area 4.67 cm^2^/well).

### Exposure of Hep and HepK6 cells to matriptase inhibitors

**Fig. 2:**
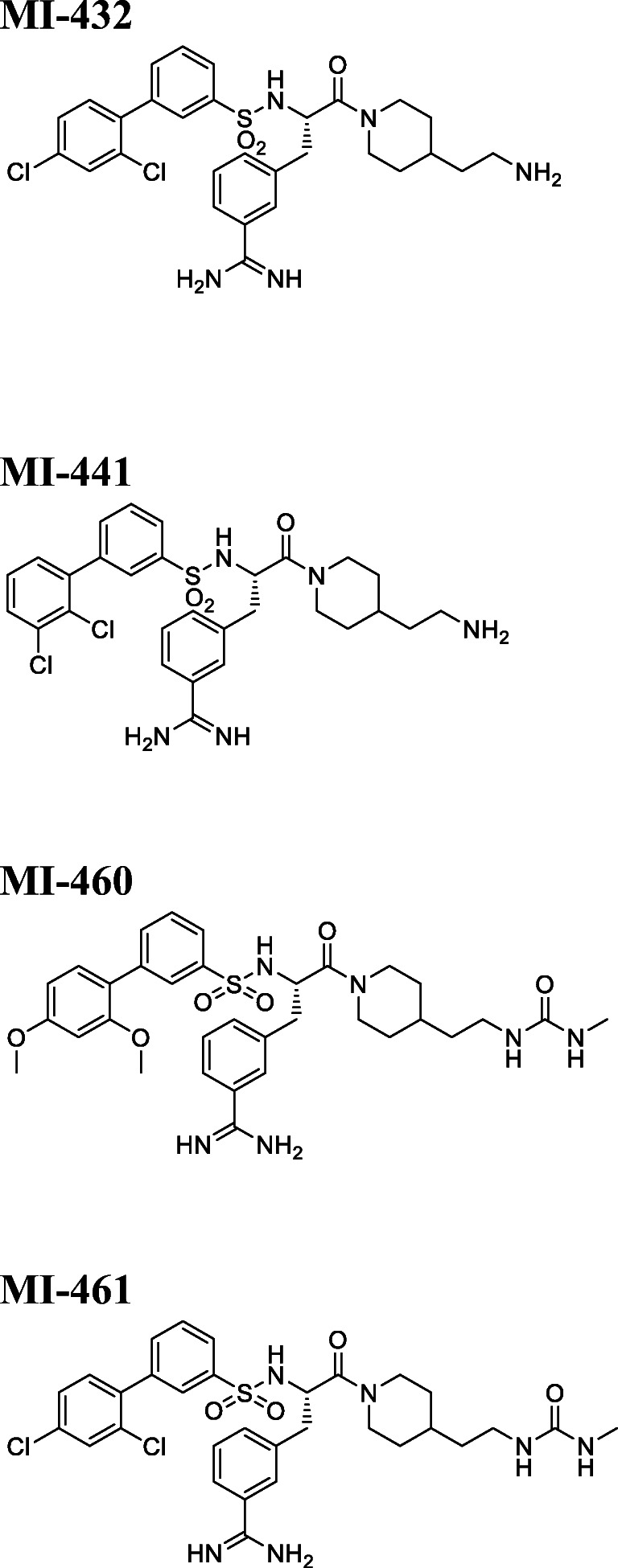
Structures of the 3-amidinophenylalanine-derived MT-1/2 inhibitors (Hammami et al. 2012) used in this study

Ten mM stock solutions of the inhibitors (MI-432, MI-441, MI-460, and MI-461) (Fig. 2) were kept at − 20 °C. Before treatment, confluent Hep and HepK6 co-cultures were washed twice with plain medium.

The solutions of the inhibitors in phenol red free Williams’ Medium E at 50 μM (in the case of MI-461, 1, 10, 30, and 50 μM) were prepared freshly prior to each experiment from the stock solutions. It was found that the selected inhibitors can be used at 50 μM concentration safely for 24 h (cell death rate was less than 5% in controls and in treated samples) in Hep mono- and HepK6 co-cultures (Pomothy et al. 2016) using MTS assay and this concentration could be applied in further experiments to characterize the effects of MT-1/MT-2 modulation in our hepatic models for 24 h. After incubation with the inhibitors for 24 h, the mono- and co-cultures were washed twice with plain medium before being subjected to the subsequent procedures.

### Hepcidin ELISA measurements

Porcine ELISA kit for hepcidin analysis was obtained from Cloud-Clone Corp. (Houston, Texas, USA). Hepcidin calibration curve was prepared using hepcidin standard solutions in different concentrations (0 pg/ml, 740 pg/ml, 2222 pg/ml, 6667 pg/ml, 20,000 pg/ml). The equation of calibration curve was the following:

Absorbance = 0.000318725 × concentration_hepcidin_ (pg/ml) + 0.0011 with *R*^2^ = 0.9963.

After 24-h lasting inhibitor treatment of the Hep and HepK6 (50 μM MI-432, MI-441, MI-460, and MI-461), the culture media from 2D and hydrogel-assisted cell matrices were collected, centrifuged (1000×*g* for 20 min) and diluted in PBS. For detailed elucidation of the concentration-dependent effect of MI-461 administration on hepcidin levels, 0, 1, 10, 30, and 50 μM MI-461 solutions were also added to Hep. Cell cultures were also exposed to LPS after 24-h cultivation to monitor changes in hepcidin production. The cells were incubated with LPS derived from *Salmonella enterica* ser. Typhimurium (1 μg/ml) (obtained from Sigma-Aldrich, Germany) for 1 h. The assay procedure was performed according to the manufacturer’s guideline. After addition of the 3,3′,5,5′-tetramethylbenzidine (TMB) substrate solution for 15 min, the intensity of color developed is reverse proportional to the concentration of hepcidin in the sample. The absorbance values were detected at 450 nm using an EZ Read Biochrom 400 microplate reader (Biochrom Ltd, UK) and normalized to protein content of the samples.

### Extracellular H_2_O_2_ measurement by the Amplex red method

Fluorescence ROS measurement of cell supernatant was based on the detection of H_2_O_2_ using the Amplex Red Hydrogen Peroxide Assay Kit (Invitrogen, Molecular Probes). To determine in which concentration range the assay can be used in quantitative manner, calibration curve was prepared using H_2_O_2_ solutions in different concentrations (0 μM, 0.315 μM, 0.625 μM, 1.25 μM, 2.5 μM, 5 μM). The equation of calibration curve was found as follows:

Fluorescence intensity = 9945.7 × concentration_H2O2_ (μM) + 5135.8 with *R*^2^ = 0.9972.

Following the exposure of mono- and co-cultures to MI-461 (50 μM, 24 h), the H_2_O_2_ concentrations in the medium were determined using a working solution of 100 μM Amplex Red and 0.2 U/ml HRP. After 30-min incubation with the dye at room temperature, the quantitative H_2_O_2_ contents were measured using a Victor X2 2030 fluorometer (*λ*_ex_ = 560 nm, *λ*_em_ = 590 nm).

### IL-6 and IL-8 determination by enzyme-linked immunosorbent assays

Previous characterization of LPS-induced changes in levels of pro-inflammatory cytokines such as quantitative analyses of IL-6 and IL-8 production in Hep mono- or HepK6 co-cultures model systems was performed, and data have already been published by Mátis et al. (2016). It was ascertained that LPS challenge of porcine Hep and HepK6 resulted in increased IL-6 and IL-8 levels in cell supernatants. After 24-h long inhibitor treatment (50 μM, MI-461), Hep mono- or HepK6 co-cultures were incubated in the medium for 24 h. Culture media were collected, centrifuged (245×*g* for 10 min), and diluted to measure IL-6 or IL-8 levels. Changes in IL-6 or IL-8 levels were determined by porcine-specific IL-6 (Sigma-Aldrich, St Louis, MO, USA) or by porcine-specific IL-8 (Invitrogen Corporation, Waltham, MA, USA) ELISA kits according to the manufacturer’s instructions. The absorbance values were detected at 450 nm using an EZ Read Biochrom 400 microplate reader (Biochrom Ltd, UK) and normalized to protein content of the samples.

### Model of matriptase-2 in complex with inhibitor MI-461

The coordinates of the previously described homology model of MT-2 (Sisay et al. 2010) were superimposed with the crystal structure of thrombin in complex with inhibitor MI-432 (PDB: 4e7r) (Hammami et al. 2012) followed by the deletion of thrombin. The C-terminal amine of inhibitor MI-432 was converted into a methylurea group present in analogue MI-461 using the builder function implemented in MOE (Molecular Operating Environment (MOE), 2013.08; Chemical Computing Group Inc., 1010 Sherbooke St. West, Suite #910, Montreal, QC, Canada, H3A 2R7) followed by an energy minimization of the inhibitor within the active site of MT-2. The minimization was performed with the MMFF94 forcefield implemented in MOE using the default parameters, whereas the structure of MT-2 was kept constant.

### Statistical analysis

For statistical evaluation, R 2.11.1 software package (2010) was applied. Differences between absolute means were evaluated by one-way analysis of variance (one-way ANOVA) with post hoc Tukey test, where data were of normal distribution and homogeneity of variances was confirmed. Differences were considered significant if the *p* value was < 0.05 marked with * (***p < 0.001).

## Results

### Effects on hepcidin secretion

**Fig. 3:**
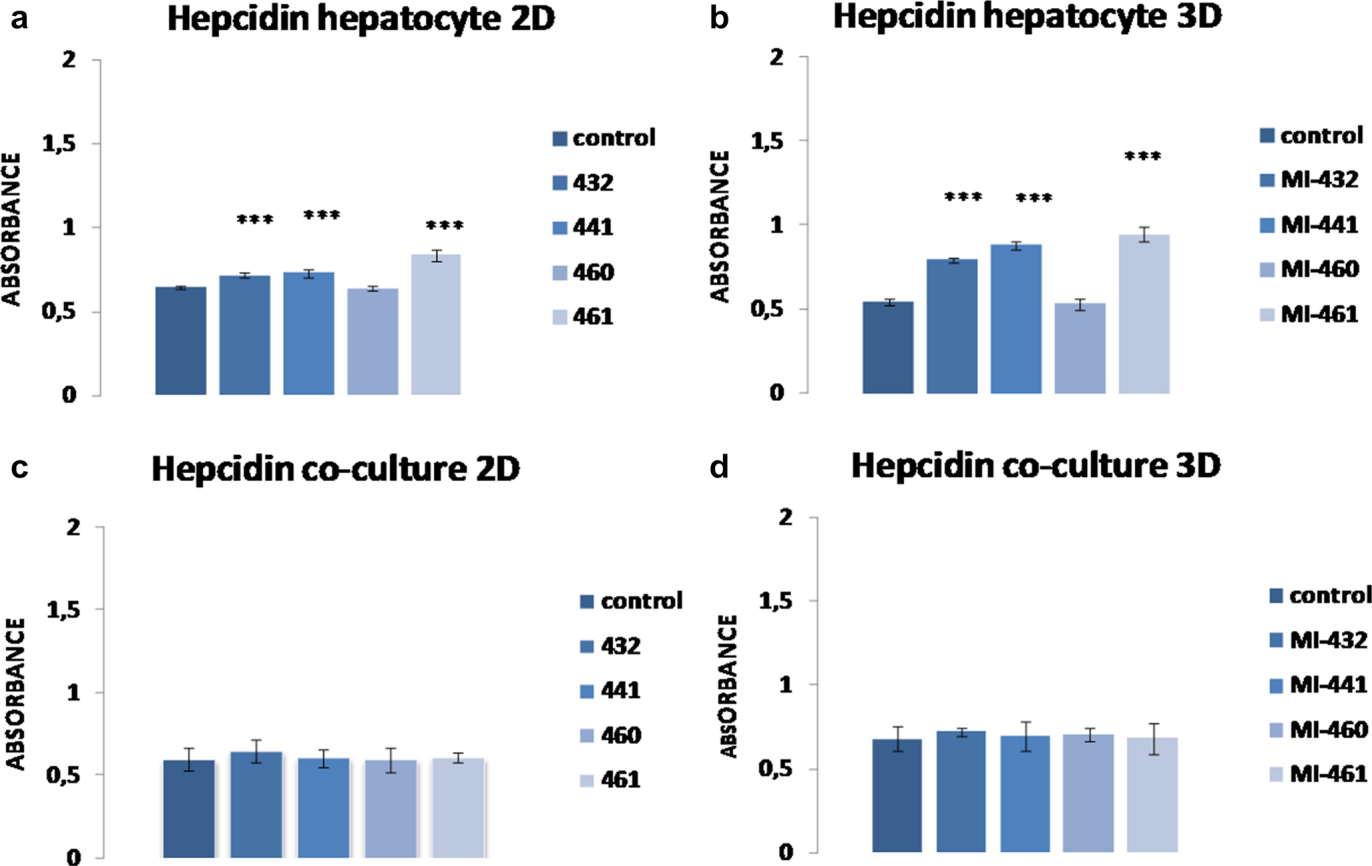
Determination of hepcidin levels of 2D and hydrogel-based hepatocyte mono- and hepatocyte-Kupffer cell co-cultures (ratio 6:1) treated with one of the four different MT-1/2 inhibitors (MI-432, MI-441, MI-460, MI-461) at 50 μM for 24 h (control in absence of inhibitors). Data are shown as average absorbance values ± SEMs (*n* = 4). **a** An increase was found in the hepcidin levels between treated and control 2D hepatocyte samples (*p* < 0.001) except in the case of samples exposed to MI-460 (*p* > 0.05). **b** Significant changes were found in the hydrogel-assisted mono-culture after MT-1/2 inhibition with MI-432, MI-441, and MI-461 (*p* < 0.001). **c**, **d** Treatment of 2D and 3D hepatocyte-Kupffer cell co-cultures with inhibitors did not result in significant increases in hepcidin production (each case *p* > 0.05)

**Fig. 4:**
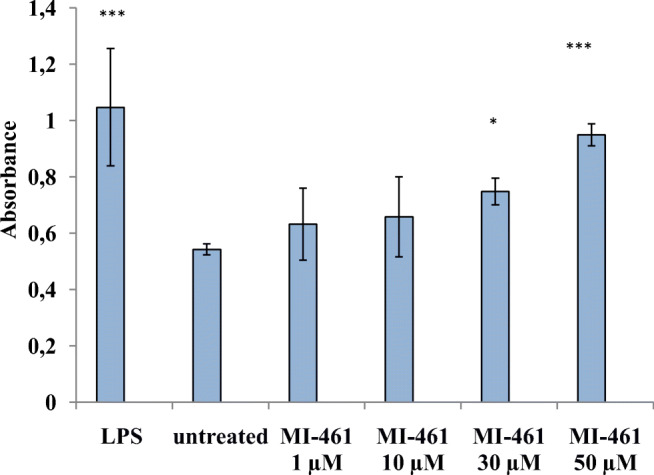
Analysis of MI-461-induced hepcidin overproduction in 3D hepatocyte samples. MI-461 was used at different concentrations (0 μM, 1 μM, 10 μM, 30 μM, and 50 μM) to elucidate concentration-dependent effect of matriptase inhibition on changes in hepcidin levels. LPS at 1 μg/ml was used as positive control (*p* < 0.001). MI-461 at 30 μM and 50 μM could induce hepcidin secretion significantly (*p* = 0.022 and *p* < 0.001, respectively). Data are shown as average absorbances ± SDs (*n* = 5)

Hep mono- and HepK6 co-cultures embedded in the presence or absence of hydrogel matrices were exposed to 50 μM of the dual MT-1/2 inhibitors (MI-432, MI-441, MI-460, and MI-461) for 24 h, and the data were compared to untreated samples (Fig. 3). In hepatocyte 2D and hydrogel-based hepatocyte 3D cultures, MI-432, MI-441, and MI-461 caused significant elevations in hepcidin secretion (*p* < 0.001) (Fig. 3a and b). In contrast, no significant changes were observed in hepcidin levels in 2D and hydrogel-assisted hepatocyte-Kupffer cell co-cultures exposed to the MT-1/2 inhibitors (*p* > 0.05) (Fig. 3c and d). It was also found that LPS at 1 μg/ml used as positive control could significantly elevate hepcidin production (*p* < 0.001). Among the tested matriptase inhibitors, only MI-461 could increase hepcidin production in vitro at 30 μM (*p* = 0.022) and 50 μM (*p* < 0.001); thus, this compound was selected for further investigations in our study (Fig. 4).

### Lack of changes in extracellular hydrogen peroxide production after MI-461 administration

**Fig. 5:**
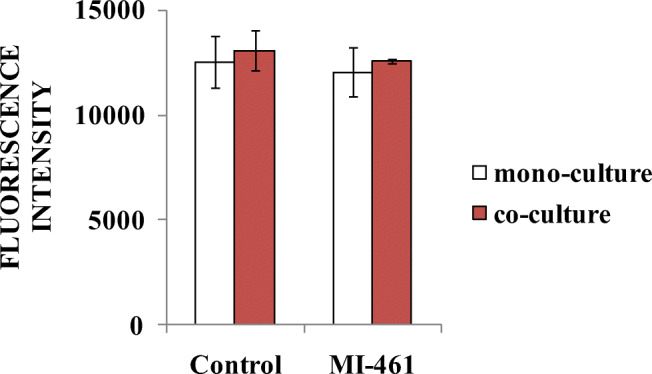
Determination of extracellular H_2_O_2_ levels by the Amplex red method after 24-h long treatment with 50 μM MI-461 on 3D mono- and co-cultures. Data are shown as average fluorescence intensities ± SDs (*n* = 3). Treatment did not cause significant differences in the hydrogen peroxide production of applied hepatic cell cultures (*p* > 0.05)

After 24-h exposition of hydrogel-based hepatocyte mono-cultures and hepatocyte-Kupffer-cell co-cultures to 50 μM MI-461, the fluorescence intensities of Amplex red were detected. No significant changes were seen in the extracelullar ROS levels in the supernatants of mono-cultures (*p* = 0.197) in the control and MI-461-treated samples. Administration of the protease inhibitor did not alter significantly the extracellular hydrogen peroxide production of the inflammatory co-culture model (*p* = 0.905) (Fig. 5).

### IL-6 and IL-8 cytokine profiles of hepatocytes and hepatocyte-Kupffer cell co-cultures

**Fig. 6:**
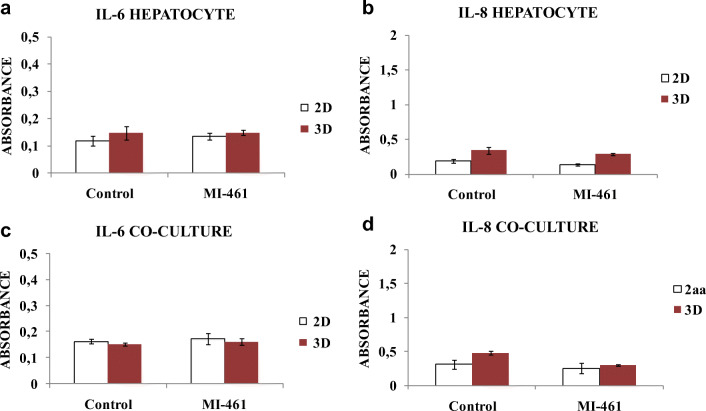
Determination of IL-6 and IL-8 levels of 2D and 3D (hydrogel-assisted) hepatocyte mono- (**a**, **c**) and hepatocyte-Kupffer cell co-cultures (ratio 6:1) (**b**, **d**) without MT-1/2 inhibition or treated with 50 μM MI-461 for 24 h. Data are shown as average absorbance values ± SEMs (*n* = 3). No significant changes were found in the IL-6 and IL-8 levels in mono-cultures and co-cultures between treated and control samples in the absence and in the presence of hydrogel-assisted matrices (*p* > 0.05). There was significant increase in IL-6 levels in supernatants of co-cultures compared to those produced in hepatocytes (*p* < 0.001) without matriptase inhibition. Regarding the cells not exposed to MI-461 treatment, no significant differences were found in IL-8 production between hepatocyte mono- and hepatocyte-Kupffer cell co-cultures (ratio 6:1) (*p* > 0.05)

IL-6 levels were not significantly altered when hepatocyte mono-cultures (Fig. 6a) and co-cultures (Fig. 6b) were exposed to MI-461 for 24 h in hydrogel-free (*p* = 0.998 in mono-cultures, *p* = 0.786 in co-cultures) and hydrogel-assisted systems (*p* = 0.934 in mono-cultures, *p* = 0.991 in co-cultures). The IL-8 levels did not change significantly after 24-h-long 50 μM MI-461 administration in hepatocytes independently of the presence of hydrogel-base (Fig. 6c in 2D, *p* = 0.053; and in hydrogel, *p* = 0.317). In hepatocyte-Kupffer co-culture system (Fig. 6d), IL-8 levels were slightly elevated compared to those in hepatocytes. No anti-inflammatory effect of MI-461 was detected (*p* = 0.051) in the hydrogel-assisted co-culture system and in co-cultures without using hydrogel (*p* = 0.823).

### Molecular modeling of the interaction between MT-2 and MI-461

**Fig. 7:**
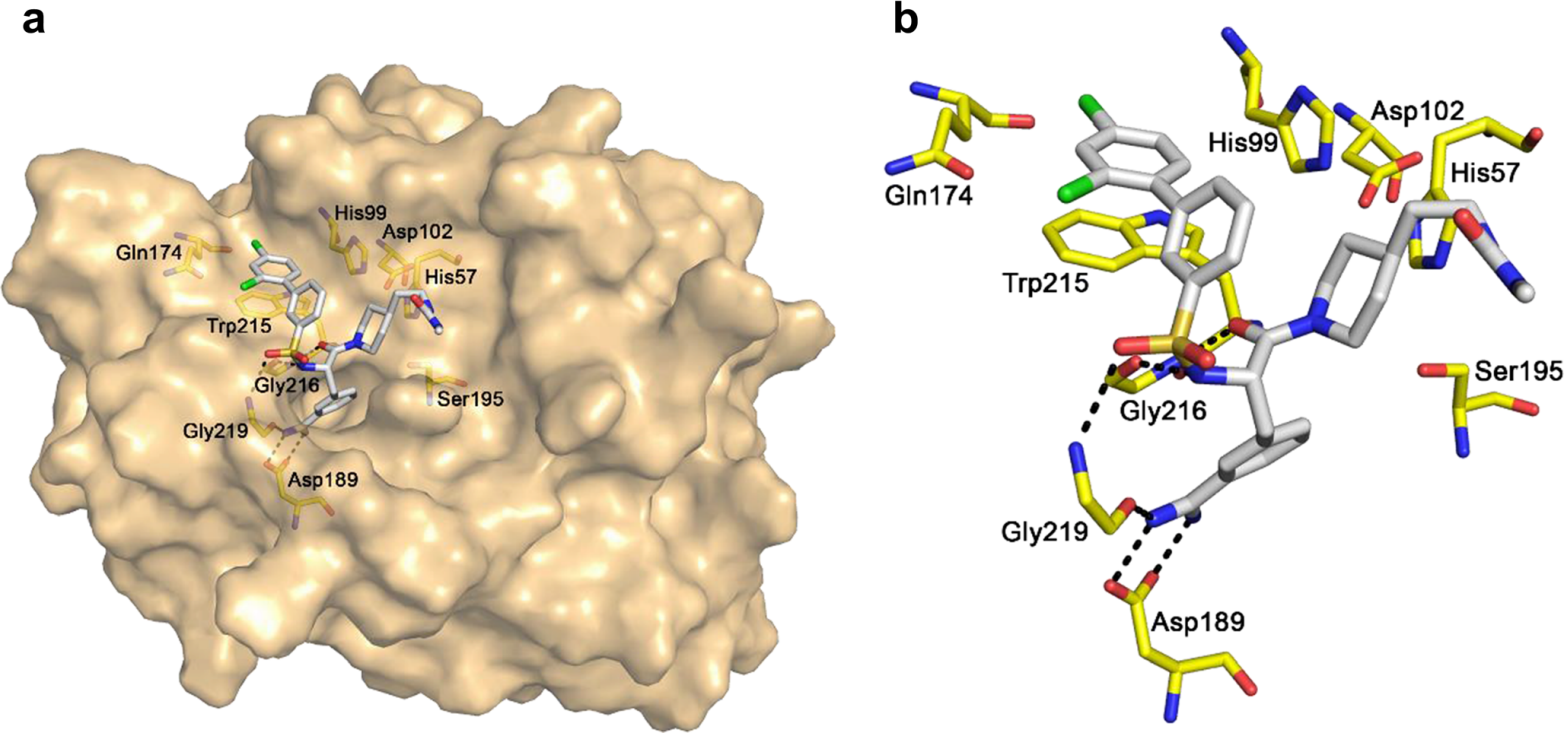
Modeled structure of inhibitor MI-461 in complex with MT-2 indicating polar interactions as dashed lines in black. Important MT-2 residues are labeled based on the chymotrypsinogen numbering. **a** MT-2 is shown with its solvent exposed transparent surface colored in beige, the inhibitor is shown as stick model with carbon atoms in white. Important MT-2 residues in the active site are given as sticks with carbons in yellow (oxygen in red, nitrogen in blue, sulfur in yellow, and chlorine in green). **b** View on the active site of MT-2 indicating polar interactions of MI-461 with residues Asp189, Gly219, and Gly216. The figures were prepared using PyMOL v0.98 (DeLano Scientific, San Carlos, CA)

When bound to the active site of MT-2, the inhibitor MI-461 adopts a very similar overall conformation as observed for related sulfonylated 3-amidinophenylalanine-piperidide derivatives in complex with various trypsin-like serine proteases such as thrombin (Bergner et al. 1995), trypsin (Renatus et al. 1998), urokinase-type plasminogen activator (Zeslawska et al. 2000), or matriptase (Steinmetzer et al. 2006). The amidine group interacts with Asp189 at the bottom of the S1 pocket and forms an additional H-bond to the carbonyl oxygen of Gly219. The backbone of the P1 residues makes an antiparallel β-sheet interaction to the NH and carbonyl of Gly216, and an additional H-bond is formed between one sulfonyl oxygen and the NH of Gly219. The N-terminal dichloro-substituted phenyl ring is accommodated in the distal S3/4 pocket above Trp215, which is surrounded by His99 on the right, but otherwise, relatively open on the left side close to Gln174. The piperidide is located in a region below His57 and His99, which is normally occupied by the side chain of the P2 residue in substrate-analogue structures. No specific interaction could be found for the C-terminal methylurea group (Fig. 7).

## Discussion

Recently, first hepcidin agonists for the treatment of iron overload have been described based on small peptidic hepcidin analogs. Moreover, analogs of BMP6, a protein which increases hepcidin expression in vivo (Andriopoulos et al. 2009), and antisense oligonucleotides targeting MT-2 activity have been developed to enhance hepcidin activities (Ganz 2013). It is worth to note that the therapeutical oral application of Mesupron, an anti-metastatic and structurally closely related 3-hydroxyamidinophenylalanine-prodrug of the active inhibitor WX-UKI-1, seems to be safe and effective in cancer treatment. WX-UKI-1 was originally described as inhibitor of the urokinase-type plasminogen activator (uPA) (Stürzebecher et al. 1999), but also inhibits MT-1 with similar potency (*K*_i_ values for uPA 0.65 μM and for MT-1 0.37 μM, respectively).

Based on our in vitro findings with hepatocytes in conventional 2D environment and hydrogel-assisted matrices, it can be reported that the applied 3-amidinophenylalanine-derived dual MT-1/2 inhibitors (MI-432, MI-441, and MI-461) at 50 μM for 24 h lead to a significant elevation of plasma hepcidin concentrations. The application of these agents might be of therapeutical importance in such diseases where too low hepcidin levels are not able to prevent excessive iron release from enterocytes, macrophages, and hepatocytes and to protect tissues from the negative consequences of iron overload.

One of these disorders is hereditary hemochromatosis, which can be formed when hepcidin expression is disturbed via mutations in the hepcidin gene itself or in the genes encoding hepcidin regulators leading to iron deposition in tissues and iron-mediated organ dysfunctions. Mutations in the genes of hepcidin or HJV can be the cause for the development of juvenile form of this disease which develops fast, and it is accompanied by severe damages including cardyomyopathy and endocrinopathies (Pietrangelo 2010; Nemeth and Ganz 2009). The hepatocyte mono- and the hepatocyte-Kupffer cell co-culture in 2D environment and embedded in a hydrogel-based 3D structures provide an excellent model system for studying the in vitro effects of synthetic protease inhibitors. Porcine hepatocyte-Kupffer co-cultures were previously used for mimicking hepatic inflammation, for studying LPS-induced inflammatory responses and for screening beneficial effects of potential anti-inflammatory agents (Mátis et al. 2016). Noteworthy, discrepancies between the effects of the selected MT-1/2 inhibitors on hepcidin productions in hepatic mono- and co-cultures may suggest different mode of actions of dual MT-1/MT-2 inhibition in the case of pathophysiological conditions.

The biochemical characterization of the matriptase inhibitor MI-461 containing an N-terminal dichlorobiphenyl-3-sulfonyl group revealed that this compound did not perturbate the redox state of Hep and HepK6 cultures based on the data from extracellular hydrogen peroxide measurements when it was used at 50 μM concentration for 24 h. It was also ascertained that MI-461 did not increase the levels of two proinflammatory cytokines, IL-6 and IL-8 levels; thus, the application of MI-461 did not provoke inflammatory responses in hepatocytes and hepatocyte-Kupffer cell co-culture systems. The administration of more efficient MT-2 inhibitors might present a novel drug alternative to prevent the excessive release of inflammatory mediators and hepatic dysfunction characteristic of iron overload in Kupffer cells (Jomova and Valko 2011; She et al. 2002).

In conclusion, our in vitro pharmacological investigations revealed that combined MT-1/2 inhibition itself does not induce oxidative stress, and it can exert beneficial effects on restoration of tipped iron metabolism via regulation of hepcidin production. The different findings between measurements performed on monolayer or on hydrogel matrices require further experiments, for example, by applying hepatocye-Kupffer cell co-culture with different cell ratios or by employing various 3D cultivation methods to investigate matriptase inhibitor-caused pharmaco-toxicological effects in-depth.

It is widely accepted that MT-2 can reduce hepcidin concentrations via cleavage of hemojuvelin. Inhibition of MT-2 leads to reduced hemojuvelin cleavage (and to higher hepcidin levels) resulting in lower iron concentration in blood. No papers have been published yet which at least assume that hemojuvelin cleavage can be accomplished via thrombin, factor Xa, and MT-1. However, with a *K*_i_ value of 0.073 μM inhibitor, MI-461 is only a moderately active MT-2 inhibitor and is still a relatively non-selective compound because it also inhibits MT-1 and other trypsin-like serine proteases. Therefore, the development of more potent and ideally also selective MT-2 inhibitors is required in the future. The model of inhibitor MI-461 in complex with MT-2, indicating several polar contacts which contribute to the submicromolar potency of this 3-amidinophenylalanine derivative, reveals that this inhibitor type is a suitable start structure for further lead optimization.
